# Some Relations of Hysteria

**Published:** 1890-03

**Authors:** J. M. Dutton

**Affiliations:** Boston, Mass.


### SOME RELATIONS OF HYSTERIA.

(Read at the Meeting of the Organon Society, Feb. 5th, 1890.)

J. M. Dutton, M. D., Boston, Mass.

The large family of functional nervous diseases seems to be constantly increasiug. Nervous prostration has become a household word, since a large proportion of the chronic cases among the women of to-day have their origin, more or less intimately, in this system of the body. In face of these facts, the point of view of the medical profession, and the ground of their work have greately changed, and in nothing do we see the revolution more markedly than in their estimation of the origin and importance of hysteria. To call a patient hysterical forty years ago meant, in the minds of most physicians, that she was silly or shamming or could get well if she pleased. To-day hysteria has taken its place as "the great neurosis" among the obscure diseases of the nervous system, and the expression of a diathesis which later may develop into an incurable disease.

Believing, theoretically at least, that Homœopathy includes all the scientific work of allopathists, with the addition of a consistent and curative system of therapeutics, it seems time well spent to review briefly some of the scholarly work done recently on the subject of hysteria by the master-minds in the old school of medicine. Volume VI of the *Index Catalogue of the Library of the Surgeon-General's Office*, contains a Bibliography of Hysteria of seventeen double-column pages. The references are to three hundred and eighteen books and nine hundred and fourteen journals. As homœopathic physicians, can we learn anything from this mass of literature, or does the mildew of materialism, which searches endlessly among decaying cells for a departed spirit, blight it all for us? Fortunately, any pathology of hysteria is, by the confession of the old-school men, entirely lacking. They have, however, made several guesses at the probable state of the nerve-tracts during hysterical manifestations which seem worthy of note. Dr. Chas. K. Mills, a voluminous writer on nervous diseases, in an interesting article on Hysteria in *Pepper's System of Medicine*, gives two hypotheses. First, that the phenomena are due to changes in the vaso-motor system. The irritability of the nerves which control the calibre of the blood-vessels in neurotic people is shown by a large number of symptoms, among them chills, cold hands and feet, and a tendency to fainting. In syncope the higher cerebral functions are superseded, presumably through a contraction of the blood-vessels of the brain cortex. In favor of this hypothesis is a case of right-sided hemianæsthesia. The special senses were affected, the sight of the left eye being almost wanting. Ophthalmoscopic examination showed the fundus of the right eye to be normal, while the left gave evidence of a contraction of the retinal blood-vessels to one-third their normal calibre. The curious researches of Anjel on the peripheric blood flow, during brain activity, give experimental evidence of vaso-motor changes in the brain. In normal persons, during mental activity, the turgescence of the tissues of a limb, inclosed in a plethysmograph, is found to diminish, presumably from the afflux of blood to the brain, but in neurasthenics, under the same circumstances, the plethysmograph registers no change.

That this well-known vaso-motor hypothesis does not cover the whole ground Dr. Mills allows. It is interesting to us, as homœopathists, to find his second hypothesis to be the dynamic origin of the disease. Of this he makes very little. Indeed, he confesses that it is equivalent to saying we know nothing about it as yet.

Dr. Mary Putnam Jacobi considers the condition fundamental to hysteria to be a congenital or acquired deficiency in the power of nerve elements to affect the storage of force in nerve tissue. The centres connected with the nerves of special senses and of common sensibility are, from the beginning of life, exposed to the most incessant stimulation from constant impressions from the outside. The storage capacity of the sensory centres is not deficient. It is rather the portions of the nervous system associated with the liberation of energy in action. These energies are of two kinds—motor and mental. The stimuli which provide for the storage of force-material in the nerve-centres concerned in mental and in motor action are far more indirect than the stimuli to sensory centres. It seems to be the discharge of motor energy which, possibly by emptying the cell of a certain amount of material, determines the gain of new material from the blood, and its storage in forms of higher complexity. It is evident that the nutrition of the muscular fibre depends largely upon muscular action, which decomposes and eliminates the material from the storage-cells. Where volition is involved there exists the possibility of avoiding action and so lessening the amount of stimulus which is necessary to the motor mechanism. This theory of deficient storage force in hysteria is confirmed by the inability, as compared with persons soundly organized, to bear fatigue, mental exertion or emotion, privations of food or air, peculiarities noticeable even in persons who at the time seem in good health, with these constitutional tendencies latent.

Sir James Paget, in an interesting series of articles on "Nervous Mimicry of Organic Disease," proposes to separate cases of simulated hip-joint and spinal disease, paralysis, contractures, etc., substituting neuromimesis for hysteria, as a name better expressing the condition. "Hysterical patients," as he tersely puts it, say "I cannot," it looks like "I will not," it is "I cannot will."

This is only partially true. A true paralysis of the will occurs in non-hysterical people and has recently been studied by alienists.

Sir James Paget is not alone in attempting to rid the world of the title "hysteria"—a word whose derivative preserves to us the absurd misconceptions of former ignorance—but none of these have been accepted, nor have passed into practical parlance. Dr. Beard's well-known essay on "Nervous Exhaustion," was written in 1868. He then first used the word "neurasthenia," which has since been so generally adopted. He distinguishes this, however, from hysteria, and his lead is usually followed in later articles. The only distinction between them, however, which is invariable and does clearly separate between them, seems to be the sex and temper of the patient. If this be a female and notably weak and selfish the case is pronounced hysteria. If a man or a woman of natural force of character it is called neurasthenia. This seems hardly a fair distinction, since marked intellectual ability, even genius, are often found in hysterical patients. Madame de Stael indulged in the most violent outbursts of hysterical emotion, Charlotte Brontê suffered from prolonged hysterical hypochondria, George Eliot was the victim of hysterical headaches and minor forms of the disease. Burton, the author of *The Anatomy of Melancholy*—a monument of curious learning and quaint wisdom—was himself a victim of the gloomy mood he so well dissected. Mahomet had fits, which do not seem to have been epileptic, which were accompanied by depression of spirits, trembling, and a sort of trance, with a strong tendency to suicide.

Reference has been made to the belief among many physicians that hysteria is the expression of a diathesis, more particularly of scrofulosis and tuberculosis.

To followers of Hahnemann this seems like a truism. The expression in the old school of so much insight into the causes and relations of disease is, however, rare enough to be interesting. Grasset, the Professor of Practice in the Montpellier School of Medicine, writing in 1884, gives utterance to the following opinions: "Tuberculosis, like all other diathesis, is an essentially general and constitutional disease of which pulmonary phthisis is but one manifestation. It may show itself as a neurosis, more particularly as hysteria. In speaking of hysteria as a tuberculous disease, therefore, we do not assume the existence of tuberculous matter is the nerve centres, for we do not look upon tubercles as pathognomonic of the diathesis, which exists without them, as they may exist without it."

He then carefully tabulates forty-four cases. In twenty-five the neurosis was the only manifestation of the disease, other members of the family suffering from phthisis. The remaining nineteen show an alternation of symptoms, and Grasset proceeds to demonstrate how hysteria often moderates phthisis, phthisis in neurotic subjects having slow development and periods of long intermission. He adds that the Montpellier School of Medicine sanctions and supports his point of view.

This is satisfactory, since with unimportant exceptions the article might have been written by a disciple of Hahnemann.

There remains the discussion of the relations of hysteria to insanity.

Sir James Paget believes that a large majority of the worst cases of nervous mimicry occur in families in which insanity has been frequent. He believes the relation of hysteria and hypochondria to insanity is often more one of degree than difference in kind. He considers that, whatever insanity may be as a disorder of some portion of the brain, the like is nervous mimicry as a disorder of other and lower nervous centres, and adds—surely any nervous centre may "go mad," as well as any part of the brain which is appropriated to the use of the mind.

Dr. Lloyd has done interesting work on this subject in an article, "Hysteria, a Study in Psychology," published in the tenth volume of *The Journal of Nervous and Mental Diseases*. The reasoning is, however, so close that it cannot be represented by extracts.

To summarize this very incomplete review of some of the work done by the allopathic school, we have considered the changes of opinion about the cause of hysteria. We then reviewed some of the hypotheses about the condition of the nervous system during the incubus of hysteria; it being possibly:

1st. A vaso-motor change.

2d. A change purely dynamic.

3d. Possibly due to a deficiency in the storage force of the motor and mental sphere, or,

4th. Due to a paralysis of the will.

We next considered the causal relations of hysteria to tuberculosis, and, lastly, its relation to insanity. Turning now from a review of hysteria, from the point of view of the old school, I will state, as briefly as may be, a case of neurasthenia treated homœopathically. It is selected because it shows the incompleteness of Sir James Paget's clever dictum, "she says 'I cannot' it looks like 'I will not;' it is 'I cannot will.'"

L. E. C., æt. thirty. Her inheritance is a peculiar one. Her mother is profoundly hysterical, and has never once in her life been told the plain truth about herself. By virtue of a hypertrophied heart she has kept her family in subjection to her sensations now forty years. The father was reserved, dignified, self-forgetful, strong of purpose, and of iron will. He is now, unfortunately for his daughter, dead. My patient has inherited both these characters markedly. Dr. Jekyll and Mr. Hyde were not in closer quarters than are these two temperaments fighting out their differences as best they may in the narrow limits of this body. Here we have what we have supposed would be the salvation of hysterical patients, could we obtain it for them, a vigorous will-power; but imagination and sensation in spite of it run riot over and about it. L. E. C.'s circumstances are these—she has to manage a house filled with boarders, her mother's sensations and her own, which are an army in themselves. My notes of her symptoms date back four years. She came to me, drenched with allopathic drugs—the bromides, iron, arsenic, brandy every day. The report of her mental symptoms I copy from my note-book: "Greatly worse from the presence of people. Before going down-town the terror and apprehension she suffers bring on vomiting and diarrhœa, some times two or three times. Never eats breakfast when going out in the morning. Believes herself pursued, always walks near the building to protect one side, must keep constantly in motion, because only thus can she distance the enemy. Almost unable to sit quietly in a horse-car, the people are so terrible. Uses all the strength she has not to scream and run away. Comes out of the cars trembling and in a cold perspiration from the effort. Cannot go into public assembly; the enemy gets up with her and pulls from behind, the seat moves from under her and she thinks herself pitching forward." Remember, this woman never did scream or run away once. She went down-town, into crowded streets when it was necessary, she quieted and controlled her mother and smoothed the ruffled tempers of the boarders, at what cost no one but herself will ever fully know. Among physical symptoms were menstruation so profuse as to leave her, after ten days, exsanguinated and greatly weakened; constant dyspepsia with nervous nausea. As a child she had had croup and suffered now frequently from acute attacks of spasmodic strictures of the throat which the old-school physicians had called laryngismus stridulus. This was the picture of the case as I first saw it. Now, after four years' avoidance of drugs and frequent homœopathic treatment, the condition is this: Not well, she never will be until rid of the incubus of her mother, but greatly relieved. No attacks of stricture of the throat since eighteen months; menstruation painless, usually normal in amount; mental symptoms generally same, with occasional relapses which give a faint impression of this disorder formerly drawn in with such black lines. This usually silent, reserved woman broke out recently into exclamations of praise and gratitude to Homœopathy. It had made the world over for her, it had made life endurable.

To describe the remedies which have brought about this happy result is difficult. Under the wisest and most experienced application of remedies the way would have been winding, for no one remedy could probably have made a cure, and I had scant experience in the use of the homœopathic remedy when I began with the case. Not to confuse matters, then, I will give one prescription:

Dec. 28th, 1886, three months after beginning treatment. "Vertigo, with faint nausea on turning to the left; even one inch from the median line would bring it on. This symptom was much emphasized. Great terror at the approach of people, must keep in motion, depressed during the effort of digestion, yet has the impulse to eat, frequently faint, gone, needs food. Complexion very pale and sallow. Menses like a hemorrhage violently profuse. Iodine$^{cm}$."

Jan. 18th, record reads: "last prescription serious mistake. Patient much worse, strange, yellow pallor of skin. Dislike of people intensified, says: 'I dare not look at any one because I know hate is in my eyes.' Thoughts of suicide. Tedium vitæ pronounced. Is menstruating. Is surprised that the flow is so small, thick, black; never so before; no pain." I gave nothing, not from an intelligent appreciation that I had the right remedy, but from sheer fear of doing the wrong thing, and because I could see nothing to do. Improvement came slowly; nothing more was given during two months.

I now believe the Iodine was one of the most important agents in bringing about improvement. The curious left-sided vertigo did not return for a year, and then was far less marked. The terror of people has substantially never returned. I was led to Iodine in the attempt to cover the singular symptom, vertigo, with sick faintness, on turning to the left. Iodine was the only remedy I could find which had this. Then, it seemed to me, no remedy had so many of the peculiar mental symptoms—*e. g.*, fear of people.

Fresh from the study of diseases under allopathic care, accustomed to the treatment, which, like a bow drawn at a venture, often misses entirely, and never hits the bull's-eye with such exactitude, I was bewildered with the results obtained. In looking back and recalling my sensations I seem like a child who should set a match to a powder magazine not expecting any great effect.

The subject on which I need instruction, and which I would like to present for discussion, is this: Was the aggravation necessary to the improvement? Is the patient too sensitive to endure a CM potency?

These aggravations have been unfortunately frequent in the treatment of this patient. They certainly are not a pleasant cure, and I question whether in a case of melancholia, with a suicidal tendency, they might not be dangerous.

### DISCUSSION.

Dr. Wesselhœft—The theory advanced by Dr. Jacobi seems the most satisfactory, that certain storage-houses of nerve force are weak, and that others are overstocked. The other theory that was interesting was: "Why should not a nervous centre go mad?" It is a very bright thought. These patients are strong in some ways and weak in others. They often have the finest perceptions and the greatest intelligence, and you will often see self-sacrificing lives shown in these neurasthenic patients when you least expect it.

In regard to the prescription of Iodine, and the patient in the next two weeks being much aggravated in her mental condition and improved in her physical symptoms, there was no doubt about not interfering, especially as the remedy was satisfactory.

As regards aggravation in general, my own experience has been that I have seen more of them in the last ten or fifteen years than before, owing either to better observation or to a longer time being given to the selection of the remedy. The aggravations have been more intense, but the cures have been better, and the remedies have been allowed to act longer. I have now a case in which Causticum has been given in the CM, DM, and CMM potencies at intervals of not less than ninety days. I used to repeat oftener, and did not respect the remedy. The long-continued aggravation of two weeks is rather rare; usually, in my experience, it is from four to ten days.

Dr. Tompkins—You spoke of mental aggravation and improvement in the physical condition, did you mean this as a special indication that the remedy was acting well?

Dr. Wesselhœft—Usually the mental symptoms are ameliorated first.

Dr. Dutton—Would an aggravation have come from the 200th?

Dr. Wesselhœft—That is a question that can only be settled by a long series of observations.

S. A. Kimball, M. D., *Secretary.*

